# Rationale for Intervention and Dose Is Lacking in Stroke Recovery Trials: A Systematic Review

**DOI:** 10.1155/2018/8087372

**Published:** 2018-10-30

**Authors:** Karen Borschmann, Kathryn S. Hayward, Audrey Raffelt, Leonid Churilov, Sharon Kramer, Julie Bernhardt

**Affiliations:** ^1^Florey Institute of Neuroscience and Mental Health, University of Melbourne, 245 Burgundy St., Heidelberg 3084, VIC, Australia; ^2^NHMRC CRE Stroke Rehabilitation & Brain Recovery, 245 Burgundy St., Heidelberg 3084, VIC, Australia; ^3^Brain Behaviour Laboratory, Department of Physical Therapy, The University of British Columbia, Koerner Pavilion UBC Hospital, 2211 Wesbrook Mall, Vancouver, BC, Canada V6T2B7

## Abstract

**Background:**

The ineffectiveness of most complex stroke recovery trials may be explained by inadequate intervention design. The primary aim of this review was to explore the rationales given for interventions and dose in stroke rehabilitation randomised controlled trials (RCTs).

**Methods:**

We searched the Cochrane Stroke Group library for RCTs that met the following criteria: (1) training based intervention; (2) >50% participants who were stroke survivors; (3) full peer-reviewed text; (4) English language. We extracted data on 16 quality items covering intervention dose (n= 3), trial design (n= 10), and risk of bias (n= 3) and 18 items related to trial method. Logistic regression analyses were performed to determine whether (1) reporting of trial quality items changed over time; (2) reporting of quality items was associated with the likelihood of a positive trial, adjusted for sample size and number of outcomes.

**Results:**

27 Cochrane reviews were included, containing 9,044 participants from 194 trials. Publication dates were 1979 to 2013, sample size was median 32 (IQR 20,58), and primary outcome was reported in 49 trials (25%). The median total quality score was 4 (IQR 3,6) and improved significantly each year (OR 1.12, 95% CI 1.07, 1.16, p<0.001). Total quality score was not associated with likelihood of a positive trial, but trials containing a biological rationale for the intervention were more likely to find a difference in patient outcome (OR 2.18, 95% CI 1.14, 4.19, p=0.02).

**Conclusion:**

To develop breakthrough treatments we need to build the rationale for research interventions and testing of intervention dosage. This will be achieved through a collective research agenda to understand the mechanistic principles that drive recovery and identification of clearer targets for clinical trials.

## 1. Introduction

Understanding acute stroke biology, critical time windows, and careful patient selection has transformed acute stroke treatment and improved patient outcomes. This did not happen overnight [1] but progress is now rapid. While the methodological quality of randomised controlled trials (RCTs) in postacute stroke rehabilitation has improved over the past 40 years [2, 3] our intervention toolkit remains bereft of treatments that markedly alter expected recovery trajectories. The relationship between trial quality and outcomes is not self-evident, since a clear, positive relationship between lower trial quality and positive outcomes has been shown in both preclinical [4] and clinical research [5]. As our attention increasingly turns to the field of stroke recovery, the international community is focused on the development of breakthrough recovery treatments [6, 7]. Beyond the logistical challenges and costs inherent in conducting complex rehabilitation and recovery trials [8] developing suitable methods and improved reporting are critical steps in this process [7–9] with specific recommendations recently published in several key areas [10–14].

Over the last few years and in response to a number of meta-analyses suggesting higher dose (amount) of motor interventions is associated with better recovery [16–19] it has been tempting to blame neutral results in large and small trials on "insufficient dose" [20]. While authors of a recent review suggest that no amount of training changes outcomes [21], we know that high doses of motor interventions early after stroke can lead to poorer outcomes [22]. Within this context, we felt it was timely to review the attention that rehabilitation trialists have given to developing the rationale for interventions in clinical trials, to put a line in the sand so to speak, before new recommendations for intervention development and reporting hopefully take effect [23]. Therefore, the primary aim of this review was to explore the rationale given and reporting of interventions and dose in stroke rehabilitation RCTs over time. A secondary aim was to examine the association between trial quality and positive trial results. Given the volume and exponential growth in rehabilitation research over the past 10 years (13,477 Medline 2012-2016, for search strategy please see Supplementary Table 1), a systematic review with data extraction from all RCTs was simply unfeasible. We elected to limit this review to RCTs that had already met inclusion for Cochrane Systematic Reviews of training based rehabilitation (motor, speech, and cognitive) interventions where researchers studied two or more intervention doses.

## 2. Methods

This review follows the PRISMA reporting guidelines [24]. We searched the Cochrane library (http://stroke.cochrane.org, September 2013) for reviews with RCTs that met the following criteria: (1) evaluation of a training based intervention, (2) at least 50% of participants who were stroke survivors, (3) full text article published in a peer-reviewed journal, and (4) available in English. We excluded trials of pharmacological agents, nutritional supplementation, surgical interventions, or therapeutic devices that were not embedded in physical training programs.

Two researchers (KB; AR) screened titles or abstracts of the Cochrane reviews and their included RCTs to determine whether individual RCTs met inclusion criteria, with a third adjudicator (JB) consulted if necessary. If any selected Cochrane review had been updated prior to data extraction, the most recent review was included. Data were extracted by one of six researchers (AR, KB, SK, KH, HW, and JB). We examined interrater reliability by double rating 40 trials (20%).

Because no existing tool met our objective of exploring the rationale for intervention design and dose, we developed a composite data extraction tool with 16 items that included: intervention dose (n= 3, based on the TIDIER guidelines [12]), trial design (n= 10, based on Medical Research Council recommendations [13]), risk of bias (n= 3, taken from the Cochrane reviews' risk of bias tool), and 18 trial methods items that are included in Table 1. "Positive trial" was defined as a significant difference between groups on the primary outcome after intervention, in favour of the intervention group. Definitions of key terms came from existing literature or group consensus when no definition was available.

## 3. Data Analysis

Descriptive statistics were used to summarise trial characteristics. Collinearity was tested between items related to trial design (e.g., hypothesis stated; reporting of adverse events) and intervention dose (e.g., justification of dose, reporting of dose intensity). Values were within acceptable limits. “Total quality score” was calculated as count (without hierarchy) of the number of quality items addressed in each paper (i.e., total score was out of 16).

Two logistic regression analyses were performed. The first was to investigate whether reporting of trial quality items changed over time. In these analyses, individual quality items were dependent variables and year of publication was the independent variable.

In the second analysis, under the caveat that the primary outcome was prespecified at data extraction by our researchers if it was not reported in the manuscript, we performed an exploratory analysis of the whether trial design factors were associated with the likelihood of a positive trial. In these models, "positive trial" was the dependent variable with each of the 16 quality items input individually as independent variables to predict associations. Models were adjusted for study sample size and number of outcomes assessed, given that these items will influence effect size and therefore the likelihood of finding a positive effect.

Analyses were performed using STATA v 13 IC statistical software (StataCorp LP, College Station, Texas, USA). A significance level of* p=* 0.05 was set for all statistical tests and no correction for multiplicity was undertaken due to the exploratory nature of this analysis [25].

The interrater reliability of data extraction by two researchers was assessed using Cohen's Kappa coefficient on the 40 trials. Levels of agreement were rated as poor< 0.00, slight 0.00 – 0.20, fair 0.21–0.40, moderate 0.41–0.60, substantial 0.61–0.80, and almost perfect 0.81–1.00 [26].

## 4. Results

### 4.1. Included Trials

**Figure 1:**
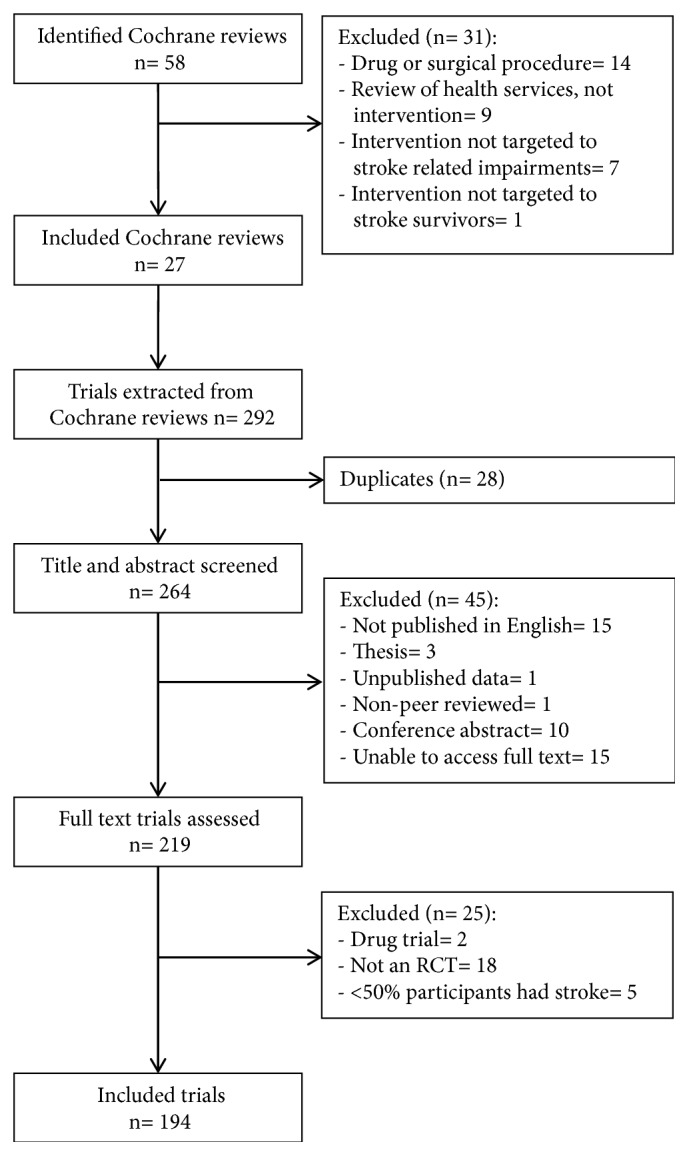
Flow diagram of identification and inclusion of Cochrane reviews and individual trials.* Note*. RCT= randomised controlled trial.

Fifty-eight Cochrane stroke rehabilitation reviews were identified and 27 met inclusion criteria, Figure 1. Included Cochrane reviews contained 292 trials, of which 28 were included in more than 1 review, and 70 did not meet inclusion criteria. The 194 included trials comprised 9,044 participants. Details of included Cochrane reviews are shown in Supplementary Table 2, and characteristics of trials included in this review are displayed in Table 2. Most trials (n= 183, 94%) were published after the 1996 release of the Consolidated Standards of Reporting Trials (CONSORT) guidelines for reporting of RCTs [10]. Half of the trials (n= 101, 52%) were published after July 1 2005, the date that compulsory trial registration was mandated for International Committee of Medical Journal Editors (ICMJE) publication [27].

### 4.2. Interrater Reliability

The kappa agreement for extracted quality items was >60% (ranging from substantial to almost perfect agreement), except for "dose intensity reported", which showed moderate agreement (55%).

### 4.3. Trial Development, Design, and Quality

Half (99/194, 51%) of trials were positive. Publication dates ranged from 1979 to 2013, with the likelihood of trials being positive increasing each year (OR= 1.05, 95% CI 1.01-1.10, p=0.018), Table 3. Sample size was typically small (median 32, IQR 20 to 58) and did not change over time. Trial phase classifications based on Medical Research Council guidelines were: development (n= 21, 10.8%), feasibility/ piloting (n= 135, 69.6%) and evaluation (n= 38, 19.6%). Piloting of the intervention was reported in 43 trials (22.2%), of which 30 (69.8%) had been published. The likelihood of pilot testing did not change over time. Stratification of participants by level of impairment, age, sex, stroke hemisphere, functional status, mobility status, or study site occurred in 42 trials (21.6%) and was more common in larger trials (OR 1.02, 95% CI 1.01, 1.03, p< 0.001).

The primary outcome measure was specified in only 49 trials (25%), and of these, the timing of the primary outcome assessment was stated (or there was only one assessment after intervention) in 27 trials (55%). Over time, per year, it was increasingly likely that study authors specified a primary outcome (OR 1.21, 95% CI 1.11, 1.33, p<0.001). The median number of outcomes assessed per trial was 4 (IQR 3 to 8). There was a single outcome measured in 10 trials (5%), and the maximum number was 28. There were 139 different outcome measures specified, and of these, only 45 (33%) were used in more than one trial. Walking speed was the most commonly assessed outcome (n= 15, 7%).

The median total quality score was 4 (IQR 3, 6), and trial quality improved significantly each year (OR 1.12, 95% CI 1.07, 1.16, p <0.001). Total trial quality was not associated with likelihood of a positive trial (OR 1.04, 95% CI 0.92, 1.19, p= 0.51). Associations between positive trial and the 16 individual characteristics of trial quality are reported in Table 3. The biological rationale for the intervention was described in only 60 trials (31%), but the inclusion of a biological rationale was associated with increased odds of a positive trial (OR= 2.18, 95% CI 1.14, 4.19, p=0.02).

### 4.4. Rationale and Scheduling of Intervention Dose

**Figure 2:**
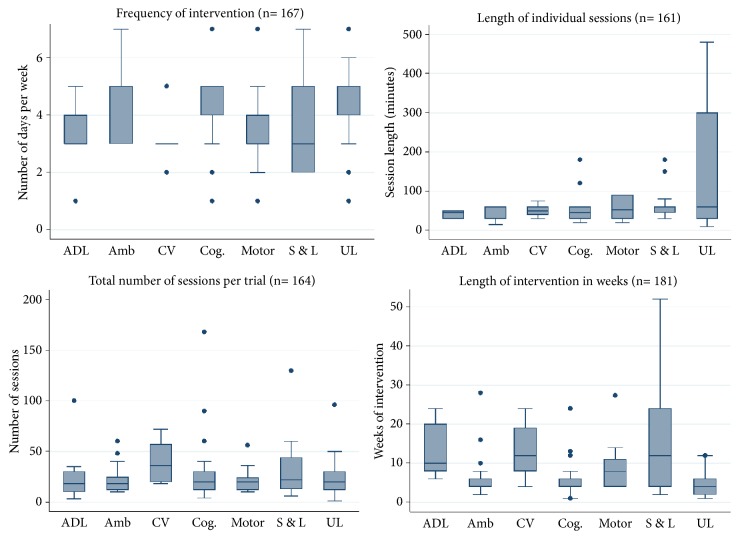
Dose schedule by intervention type* Note*. N in graph heading= number of trials that contained a report of this item. Type of intervention: ADL=activity of daily living, amb= balance or mobility, cog.= cognition or neglect, CV= cardiovascular or strength, motor= motor control, S & L= speech and language, and UL= upper limb.

Interventions commenced at a median 142 days (IQR 32, 815) after stroke. In only 11 trials did the intervention commence within 7 days of stroke, and an additional 31 started within 30 days. The median dose schedule across all trials was 50 minutes per session, 5 days per week for 6 weeks (Figure 2). All components of the dose schedule (i.e., session length, number of sessions per day, number of sessions per week, duration of intervention, and total number of sessions) were reported in 143 trials (74%). The intensity of intervention was reported for 69 trials (36%), and a justification for intervention dose was provided in only 38 trials (20%). Reporting of the intensity of intervention dose was associated with a reduced likelihood of a positive trial (OR=0.49, 95% CI 0.26, 0.92, p= 0.02).

Most interventions were delivered individually (n= 183, 94%), with the remainder (n= 11, 6%) delivered in a group setting. Intervention dose tailoring to individuals was reported in 67 trials (35%), and dose was progressed in 65 trials (34%). The comparison group was "usual care" in 101 trials (52%), second intervention (n= 42, 22%), attention control (n= 26, 13%), sham intervention (n= 17, 9%), and waitlist control (n= 8, 4%).

## 5. Discussion

The paper by Kidwell, Saver, and colleagues in 2001 [1] showcased the significant changes in the quality, precision and focus of acute stroke trials in the 1980s and 90s that heralded the discovery of life changing acute stroke treatments. This review of stroke rehabilitation research echoes the earlier findings of Kidwell et al., suggesting we are around 15 years behind our acute stroke colleagues.

Remarkably few trials prior to 2010 were international, many were small and trial quality was generally low. Encouragingly we found small improvements in overall methodological quality over time, potentially aided by adherence to reporting guidelines and mandated trial registration, but the quality score of trials was not associated with likelihood of a positive trial. It is perhaps not surprising that we have little breakthrough in recovery trials, as we considered that the majority of trials were actually feasibility or pilot work (despite often being described as efficacy trials) rather than staged development of phase 3 studies underpinned by a strong biological rationale. This paucity of trials with well defined, justified, piloted and biologically informed interventions is a critical consideration in future trial designs.

The idea of a critical, early window in which recovery can be optimised has gained considerable ground in recent years [28–31] yet in only 22% of trials did training start within a month of stroke. Similarly, while achieving an "optimal" treatment dose (generally higher than current usual care) is believed by many, but not all [32, 33] to be the key to driving recovery, in relatively few trials was the dose of intervention, its schedule and progression well defined. The median experimental dose schedule across the trials in our review was 50 minutes per session, 5 days per week, for 6 weeks. We would argue that this closely resembled, or was even lower than, current clinical practice (i.e., it was pragmatic) rather than testing mechanistically derived hypotheses about what is needed to drive recovery [3, 7]. Interestingly, we observed that trials in which a biological rationale for the intervention was presented, no matter how simple, were more likely to find a difference in patient outcome.

Although we focused significant attention on describing intervention timing and dose in this review, we recognise that what is being tested in rehabilitation trials may be just as important as questions of how, how much and when interventions are delivered. We currently have few biological targets in stroke recovery; our challenge is to understand which biological targets are relevant to augment natural neural repair. Algorithms based on infarct volume and initial level of impairment have recently been developed in animal models, to predict the minimum threshold of rehabilitation required to “activate” recovery [29, 30]. This model demonstrates an exciting opportunity for clinical translation.

Patient selection, stratification, and tailoring of dose were seldom reported in the trials included in this review but are critical considerations in the quest for effective recovery-promoting treatments [7, 34]. Biomarkers of recovery will increasingly play an important role to guide patient selection and targeting of interventions. New guidelines for stroke recovery trials have been published that cover development, reporting, and measurement to align preclinical and clinical stroke trials [7, 23, 34–36]. Key recommendations include imaging biomarkers in preclinical and clinical studies and standardised assessment time points and measures. We encourage the stroke community to take up these recommendations.

Our choice to perform a detailed review of 194 trials from Cochrane reviews rather than more broadly review the thousands of published trials from just the past 5 years while being pragmatic is a limitation. However, we consider Cochrane reviews to be an important source of evidence synthesis in topic areas of interest and which are likely to have informed current clinical practice. Further, we could utilise independent assessments of risk of bias from these reviews. We recognise that we have more to learn from recent high quality trials [22, 37–39] that are yet to be incorporated into Cochrane reviews. To capture other items that reflect trial quality [13] and intervention reporting standards [12] we defined, standardised, and extracted additional data from the trials. While errors in data extraction are possible with use of multiple raters, interrater reliability on most items was substantial to almost perfect.

To revolutionise stroke recovery we need to understand the mechanistic principles that drive recovery and identify clearer targets [1]. We must clearly define interventions, provide clear and relevant primary outcomes [3], and consider the biological plausibility of interventions and dose. Collaborative multidisciplinary research programs need to be developed that span the complex stroke continuum and include stroke consumers and clinicians, clinical researchers and basic scientists, editors, and funding bodies [7]. Similar to our acute stroke colleagues, we can expect to have more failures before we succeed, but with clearer understandings, research approaches, and a collective agenda we will make progress. There has never been a better time to be doing stroke recovery research.

## Figures and Tables

**Table 1:** Data extraction items: characteristics of trial design, intervention dose, risk of bias, and other methodological features.

	Trial design	Intervention dose	Risk of bias
Quality Items	Based on MRC guidelines*∗* (1) Trial registered? †‡ (2) Committee oversee of trial? †‡ (3) Hypothesis stated? ‡ (4) Trial phase reported? (5) Biological rationale of intervention included? (6) Treatment compliance measured? † (7) Actual treatment participants received measured & reported? (8) Adverse events recorded? † (9) Single- or multi-site trial? (10) Primary outcome specified? †	Based on TIDIER guidelines§ (1) Intervention dose justified? (2) Dose schedule reported sufficient for replication? (3) Dose intensity reported?	Extracted from Cochrane reviews (1) Sequence generation adequate? (2) Allocation concealment adequate? (3) Risk of detection bias addressed? ||

Trial Methods	(1) Purpose of study (2) Type of comparison group (3) Was the trial international? (4) Sample size (5) Use of stratification (6) Days post-stroke at baseline (7) Was intervention piloted (if not a pilot study)? † (8) Description of intervention components (9) Use of device in intervention	(1) Individual or group treatment? (2) Individually tailored dose? (3) Dose progression? (4) Dose schedule: session length (minutes), sessions per day & per week, duration (weeks) (5) Dose matched between groups?	(1) What was primary outcome? # (2) Positive trial? # (3) Number of outcome measures (4) List of outcome measures

*Note*. *∗*Based on Medical Research Council guidelines for developing and evaluating complex interventions [13].

†If authors did not report these details, this was recorded as "no" in data extraction.

‡Based on best practice methods but not stated in Medical Research Council guidelines.

§TIDieR guidelines [12].

| |Reported in Cochrane review as "Blinding all outcomes", "Blinding: performance and detection bias", or "Blinding: outcome assessors".

#When no primary outcome was specified by authors, the outcome most closely matching the purpose of the trial was nominated by data extractors.

*Comparison group categories*: usual care, other active intervention, waitlist, sham intervention, or attention control.

*Dose intensity*: prespecified treatment target (i.e., the amount of physical or mental work) that participants attempted to reach in a given session (either uniform for all participants or individually tailored) [15].

*Dose schedule*: session length (minutes), number of sessions per day, number of sessions per week, duration of intervention (weeks), and total number of sessions.

*Positive trial*: a significant difference between groups on the primary outcome after intervention, in favour of the intervention group [1].

*Sites*: single site= hospital inpatient-based intervention conducted at a single site. Multi-site= inpatient based intervention conducted at 2 or more sites. Multi-country= studies conducted in more than one country.

*Trial phase:* research phase of randomised controlled trial—development, feasibility or piloting, and evaluation—based on the Medical Research Council Guidelines for developing and evaluating complex interventions [13].

**Table 2:** Characteristics of 194 included trials.

Trial Characteristic	n (%)
Year of publication	
<1980	1 (0.5)
1980–1989	12 (6.2)
1990–1999	29 (14.9)
2000–2009	122 (62.9)
2010–2013	30 (15.5)
Location of first author^*∗*^	
USA or Canada	66 (34.0)
Europe	48 (24.7)
UK or Ireland	35 (18.0)
Asia	25 (12.9)
Australia or New Zealand	13 (6.7)
Middle East	6 (3.1)
South America	1 (0.5)
Type of intervention	
Motor control, upper limb	42 (21.6)
Strength / fitness	41 (21.1)
Mental practice / perceptual training	28 (14.4)
Balance / gait / ambulation	27 (13.9)
Speech and language	26 (13.4)
Sensory training, upper limb	9 (4.6)
Activities of daily living (ADL)	8 (4.1)
Cognition	6 (3.1)
Sensory training, visual field	5 (2.6)
Motor control, functional recovery	2 (1.0)
Intervention sites	
Number of studies reporting intervention site/s	124 (63.9)
Single site^†^	97 (78)
Multi-site‡	26 (21)
Multi-country	1 (1)
Sample size	
Number of studies reporting sample size	194 (100)
Median sample size (IQR)	32 (20 – 58)
Intervention commencement, days post-stroke	
Number of studies reporting days post-stroke	172 (88.7)
Days post-stroke to intervention, median (IQR)	142 (32.1 – 815.1)

^*∗*^See Supplemental Table 3 for breakdown by country.

^†^ includes 31 home-based interventions, where participants were located in a single geographical region.

‡ includes 5 home-based interventions, where the study was undertaken in several regions within the same country.

**Table 3:** Change in trial quality over time and odds of a positive trial relative to trial quality.

Quality item	Quality item reported in trial *N (%)*	Association between reporting of quality item and publication year *OR per year post 1979 (95% CI), p*	Association between reporting of quality item and likelihood of a positive trial *OR (95% CI), p∗*
Risk of bias			
Allocation concealment	68 (35.1)	1.06 (1.00, 1.14), 0.09	2.04 (0.67, 6.16), 0.21
Sequence generation	58 (29.9) †	1.0 (0.85, 1.17), 0.99	3.27 (0.27, 40.2), 0.35
Detection bias addressed	90 (46.4) ‡	1.02 (0.96, 1.08), 0.55	0.83 (0.36, 1.91), 0.67
Intervention dose			
Dose schedule report	148 (76.3)	1.06 (1.01, 1.10), 0.01	1.52 (0.76, 3.09), 0.24
Dose intensity report	69 (35.6)	1.06 (1.01, 1.12), 0.02	0.49 (0.26, 0.92), 0.03
Dose justified	36 (18.6)	1.01 (0.95, 1.06), 0.89	0.59 (0.28, 1.26), 0.17
Trial design			
Hypothesis stated	71 (36.6)	1.09 (1.03, 1.15), 0.002	1.33 (0.73, 2.46), 0.36
Primary outcome stated	49 (25.3)	1.16 (1.08, 1.26), <0.001	1.22 (0.61, 2.45), 0.56
Trial phase stated	15 (7.7)	1.09 (0.98, 1.20), 0.12	0.70 (0.40, 1.24), 0.22
Registration of trial	10 (5.2)	3.32 (1.58, 6.95), 0.001	0.61 (0.37, 5.49), 0.61
Use of committees	1 (0.5)	1.33 (0.68, 2.63), 0.41	N/A §
Study sites specified	124 (63.9)	1.01 (0.96, 1.05), 0.87	0.99 (0.53, 1.84), 0.97
Biological rationale of intervention stated	60 (30.9)	1.11 (1.04, 1.17), 0.001	2.18 (1.14, 4.19), 0.02
Compliance with intervention reported	69 (35.6)	1.05 (1.01 1.10), 0.048	1.33 (0.71, 2.49), 0.37
Actual intervention received reported	22 (11.3)	1.14 (1.03, 1.27), 0.02	1.48 (0.57, 3.84), 0.42
Adverse events reported	22 (11.3)	1.09 (1.01, 1.19), 0.07	1.42 (0.56, 3.62), 0.46

OR= odds ratio.

*∗*Logistic regression adjusted for number of outcomes and sample size.

†Out of 113 trials that were scored in the corresponding Cochrane review.

‡Out of 179 trials that were scored in the corresponding Cochrane review.

§As "design committee= 1" predicted positive trial perfectly, odds ratio cannot be calculated.

## Supplementary Materials

Supplementary Materials Supplementary Table 1: search of Medline via OVID for stroke rehabilitation controlled trials 2012-2016. Supplementary Table 2: included Cochrane reviews, number of individual trials and sample sizes. Supplementary Table 3: location by country of first author of included trials.
