# Supplementary material for: Rationale for Intervention and Dose Is Lacking in Stroke Recovery Trials: A Systematic Review
Source: Stroke Res Treat. 2018 Oct 30;2018:8087372. doi: 10.1155/2018/8087372 (PMC6234440; doi:10.1155/2018/8087372)
Supplement: Supplementary Materials — Supplementary Table 1: search of Medline via OVID for stroke rehabilitation controlled trials 2012-2016. Supplementary Table 2: included Cochrane reviews, number of individual trials and sample sizes. Supplementary Table 3: location by country of first author of included trials. [file 8087372.f1.pdf]

Supplementary materials

Online supplementary Table 1. Search of Medline via OVID for stroke rehabilitation controlled trials 2012- 2016

Line	Search Term	Records
1	exp Stroke/	104259
2	(((brain or cerebral or cerebrovascular) adj3 (accident* or infarct* or stroke)) or CVA*).mp.	57908
3	((((brain* or cerebr* or cerebell* or intracran* or intracerebral or vertebrobasilar) and vascular*) or cerebrovascular*) and (disease or diseases or accident* or disorder*)).mp.	109053
4	CVA*.mp.	3423
5	exp Cerebrovascular Disorders/ or post stroke.mp.	314551
6	((brain* or cerebr* or cerebell* or intracran* or intracerebral or vertebrobasilar) and (haemorrhag* or hemorrhag* or ischemi* or ischaemi* or infarct* or haematoma* or hematoma* or bleed*)).mp.	219722
7	plegia.mp. or exp Paralysis/	77256
8	1 or 2 or 3 or 4 or 5 or 6 or 7	478211
9	exp Randomized Controlled Trials as Topic/ or randomised controlled trial.mp.	124256
10	exp Controlled Clinical Trial/	514774
11	controlled clinical trial.mp. or exp Controlled Clinical Trial/	517914
12	(randomised or randomized or randomly or random* or random).mp.	1062025
13	group*.mp.	3162420
14	(phase 1 or phase I or phase 2 or Phase II or Phase 3 or Phase III or Phase 4 or Phase IV).mp. [mp=title, abstract, original title, name of substance word, subject heading word, keyword heading word, protocol supplementary concept word, rare disease supplementary concept word, unique identifier]	132404
15	9 or 10 or 11 or 12 or 13 or 14	3877450
16	exp Neurological Rehabilitation/ or exp Rehabilitation/ or rehabilitation.mp. or exp "Physical and Rehabilitation Medicine"/	265908
17	training.mp.	322179
18	exp Exercise Therapy/	37464
19	therapy.mp.	2025576
20	(therapies or therap* or ex* or exercise or training or train*).mp. [mp=title, abstract, original title, name of substance word, subject heading word, keyword heading word, protocol supplementary concept word, rare disease supplementary concept word, unique identifier]	13713128
21	16 or 17 or 18 or 19 or 20	13786389

22	drug.mp. or exp Pharmaceutical Preparations/	2458120
23	drug therapy.mp. or exp Drug Therapy/	1194268
24	device.mp. or exp “Equipment and Supplies”/	1392931
25	22 or 23 or 24	4263296
26	8 and 15 and 21	13786389
27	26 not 25	53279
28	limit 27 to humans	41711
29	limit 28 to last 10 years	22916
30	limit 28 to last 5 years	13477

Note: search was run on 15 August 2016.

Online supplementary Table 2. Included Cochrane reviews, number of individual trials and sample sizes included in review

Cochrane Review	Trials in Cochrane Review	Trials from Cochrane Review included in current review (Number of participants)	Reason for excluding Cochrane trials in current review
Barclay-Goddard et al. 2004<br>*Force platform feedback for standing balance training after stroke*	7	6 (230)	1= Unable to source
Barclay-Goddard et al. 2011 *<br>*Mental practice for treating upper extremity deficits in individuals with hemiparesis after stroke*	6	6 (119)	-
Bernhardt et al. 2009 *<br>*Very early versus delayed mobilisation after stroke*	1	1 (71)	-
Bowen et al. 2013 *<br>*Cognitive rehabilitation for spatial neglect following stroke*	23	20 (561)	3= Unable to Source
Bowen et al. 2011 *<br>*Non-pharmacological interventions for perceptual disorders following stroke and other adult-acquired, non- progressive brain injury*	6	2 (130)	1= <50% stroke<br>1= Duplicate<br>2= Unable to source
Bradt et al. 2010<br>*Music therapy for acquired brain injury*	7	3 (118)	3= Not RCT<br>1= Not in English
Brady et al. 2012<br>*Speech and language therapy for aphasia following stroke*<br>*Some evidence of effectiveness*	39	26 (1477)	3= Not RCT<br>6= Not in English<br>3= Not full text<br>1= Unable to source

Cochrane Review	Trials in Cochrane Review	Trials from Cochrane Review included in current review (Number of participants)	Reason for excluding Cochrane trials in current review
Coupar et al. 2010 *<br>*Simultaneous bilateral training for improving arm function after stroke*<br>*Insufficient evidence to make conclusions*	18	13 (456)	5= Not RCT
das Nair et al 2007 *<br>*Cognitive rehabilitation for memory deficits following stroke*	2	1 (12)	1= <50% stroke
Doyle et al. 2010 *<br>*Interventions for sensory impairment in the upper limb after stroke*	13	9 (353)	4= Not full text
English et al 2010<br>*Circuit class therapy for improving mobility after stroke*<br>Conclusion =‘CCT is safe and effective’	6	2 (154)	1= Not RCT<br>3= Duplicate
French et al. 2007<br>*Repetitive task training for improving functional ability after stroke*	14	11 (482)	1= Not RCT<br>2= Duplicate
Hoffmann et al. 2007 *<br>*Occupational therapy for cognitive impairment in stroke patients*	1	1 (33)	-
Legg et al. 2006<br>*Occupational therapy for patients with problems in activities of daily living after stroke*	9	7 (1075)	-

**Cochrane Review**	**Trials in Cochrane Review**	**Trials from Cochrane Review included in current review (Number of participants)**	**Reason for excluding Cochrane trials in current review**
Loestcher et al. 2013 *<br>*Cognitive rehabilitation for attention deficits following stroke*	6	4 (146)	2= Not in English
McGeough et al. 2009 *<br>*Interventions for post-stroke fatigue*	3	0	1= No RCT<br>2= Drug trial
Mehrholz et al. 2011 *<br>*Water-based exercises for improving activities of daily living after stroke*	4	2 (38)	1= Not in English<br>1= Not full text
Pollock et al. 2007<br>*Physiotherapy treatment approaches for the recovery of postural control and lower limb function following stroke.*	21	6 (403)	10= Duplicate<br>2= Not RCT<br>1=No full text<br>2= Unable to source
Pollock et al. 2011 *<br>*Interventions for visual field defects in patients with stroke.*	13	5 (125)	3= Duplicate<br>2= <50% stroke<br>2= Not full text<br>1= Unable to source
Saunders et al. 2013<br>*Physical fitness training for stroke patients*	45	39 (1891)	1= Duplicate<br>1= < 50% stroke<br>1= Not in English<br>2= Not full text<br>1= Unable to source
Sellars et al. 2005 *<br>*Speech and language therapy for dysarthria due to non-progressive brain damage*	0	0	-

**Cochrane Review**	**Trials in Cochrane Review**	**Trials from Cochrane Review included in current review (Number of participants)**	**Reason for excluding Cochrane trials in current review**
Sirtori et al. 2009<br>*Constraint-induced movement therapy for upper extremities in stroke patients*	19	17 (604)	1= Not RCT<br>1= Unable to source
States et al. 2009 *<br>*Overground physical therapy gait training for chronic stroke patients with mobility deficits*	9	2 (68)	1= Not intervention<br>6= Duplicate
Thieme et al. 2012<br>*Mirror therapy for improving motor function after stroke*	14	9 (446)	2= Not full text<br>3= Not in English
West et al. 2005 *<br>*Interventions for apraxia of speech following stroke*	0	0	-
West et al. 2008 *<br>*Interventions for motor apraxia following stroke*	3	2 (52)	1= Duplicate
Winter et al. 2011<br>*Hands-on therapy interventions for upper limb motor dysfunction following stroke*	3	0	1= Duplicate<br>1= Not in English<br>1= Unable to source
TOTALS	292	194 (9,044)	

Online supplementary Table 3. Location of first author by country

Country	Number of trials included in review
Australia	11
Belgium	3
Brazil	1
Canada	17
China	1
Finland	1
France	2
Germany	15
Hong Kong	3
Ireland	2
Israel	2
Italy	8
Japan	2
Korea	3
Netherlands	10
New Zealand	2
Norway	4
Poland	1
Portugal	1
Saudi Arabia	1
Singapore	1
Sweden	3
Taiwan	15
Turkey	3
United Kingdom	33
United States of America	49
